# Regulation of chromatin organization during animal regeneration

**DOI:** 10.1186/s13619-023-00162-x

**Published:** 2023-06-01

**Authors:** Xiaohui Jia, Weifeng Lin, Wei Wang

**Affiliations:** 1 National Institute of Biological Sciences, Beijing, 102206 China; 2 China Agricultural University, Beijing, 100083 China; 3 Tsinghua Institute of Multidisciplinary Biomedical Research, Tsinghua University, Beijing, 100084 China

**Keywords:** Regeneration, Chromatin organization, Regeneration responsive enhancer

## Abstract

Activation of regeneration upon tissue damages requires the activation of many developmental genes responsible for cell proliferation, migration, differentiation, and tissue patterning. Ample evidence revealed that the regulation of chromatin organization functions as a crucial mechanism for establishing and maintaining cellular identity through precise control of gene transcription. The alteration of chromatin organization can lead to changes in chromatin accessibility and/or enhancer-promoter interactions. Like embryogenesis, each stage of tissue regeneration is accompanied by dynamic changes of chromatin organization in regeneration-responsive cells. In the past decade, many studies have been conducted to investigate the contribution of chromatin organization during regeneration in various tissues, organs, and organisms. A collection of chromatin regulators were demonstrated to play critical roles in regeneration. In this review, we will summarize the progress in the understanding of chromatin organization during regeneration in different research organisms and discuss potential common mechanisms responsible for the activation of regeneration response program.

## Background

Regeneration is a fascinating phenomenon in biology which is depicted as the restoration of damaged body parts to their original state in response to injury or diseases. Unlike mammals including humans that usually have very limited regenerative capacities, lower vertebrates such as fishes and ﻿salamanders are good at regenerating various appendages and organs. Ample evidence from regeneration-competent animals revealed that tissues or organs with high regenerative capacities tend to retain high proliferative potential (Chen et al. 2020a; Iismaa et al. 2018; Jopling et al. 2010; Ryoo and Bergmann 2012). Like organ development, cell proliferation and differentiation are essential processes for successful regeneration of damaged organs (Tanaka and Reddien 2011). Upon tissue damage, the cell source for regeneration can vary from organ to organ. For example, progenitor cells, reserve stem cells, or terminally differentiated cells that can undergo de-differentiation or trans-differentiation are common cell sources involved in regeneration (Merrell and Stanger 2016). In classic epimorphic regeneration (*e.g.*, limb regeneration, fin regeneration, and planarian head regeneration), a series of key steps including inflammation response, re-epithelialization (wound healing), blastema formation, regenerative outgrowth, and re-patterning occur to restore the original tissue function (Londono et al. 2018; Pfefferli and Jazwinska 2015; Reddien 2018; Yokoyama 2008). The progression of each phase of regeneration requires precise regulation of gene expression.

Protein-coding and non-coding genomic DNA of each cell is well-organized inside microscopic nuclei as chromatins. The unique structure of the chromatin efficiently packages the genome without compromising DNA accessibility for proper gene expression and replication of the genetic material during cell division. The three-dimensional (3D) genome organization can be defined and characterized at different levels: the chromosomal (distinct distribution of chromosomes in the nucleus) and sub-chromosomal levels (the compartmentalization of chromatin) (Dekker and Mirny 2016; Van Driel et al. 2003; Woodcock and Ghosh 2010). A fundamental unit of the 3D-chromatin organization is the topologically associating domains (TADs) (McArthur and Capra 2021). TADs and their corresponding TAD boundaries within a given cell participate in gene regulation by facilitating or restraining interactions between regulatory sequences and targets. A lot of studies have demonstrated that the organization of accessible chromatin in a genome encodes a network of potential physical interactions that involve promoters, enhancers, insulators, and chromatin-binding factors (Klemm et al. 2019). Precise regulation of chromatin organization is essential for establishing and maintaining cellular identity. For instance, the Polycomb repressive complex PRC1 functions as a master regulator of genome architecture in mouse embryonic stem cells by constraining developmental transcription factor genes (*e.g.*, Hox genes) and their enhancers in three-dimensional interaction networks (Schoenfelder et al. 2015). It was proposed that the selective activation of genes from such a network controls cell fate specification during early embryonic development. In contrast, abnormal regulation of chromatin organization can cause developmental defects and pathogenesis (Anania and Lupianez 2020; Schoenfelder and Fraser 2019; Ushiki et al. 2021; Zheng and Xie 2019).

The regulation of organ regeneration and development share common features in many aspects (Efroni et al. 2016; Goldman and Poss 2020; Malloch et al. 2009). Dynamic changes of chromatin accessibility, epigenetic modification, and the activation of gene promoters and *cis*-regulatory elements that are known to be critical during development also play essential roles in the activation and progression of regeneration. In the past decades, fruitful new knowledge has been accumulated in the understanding of regeneration due to fast advancement in genetic tools and technologies of various fields, which allows for an easier, faster, and deeper examination of fundamental questions. In this review, we will focus on new findings regarding the regulation of chromatin organization during regeneration in different organisms and discuss potential common mechanisms underpinning the activation of the regeneration program.

## Clues from development and diseases

**Fig. 1:**
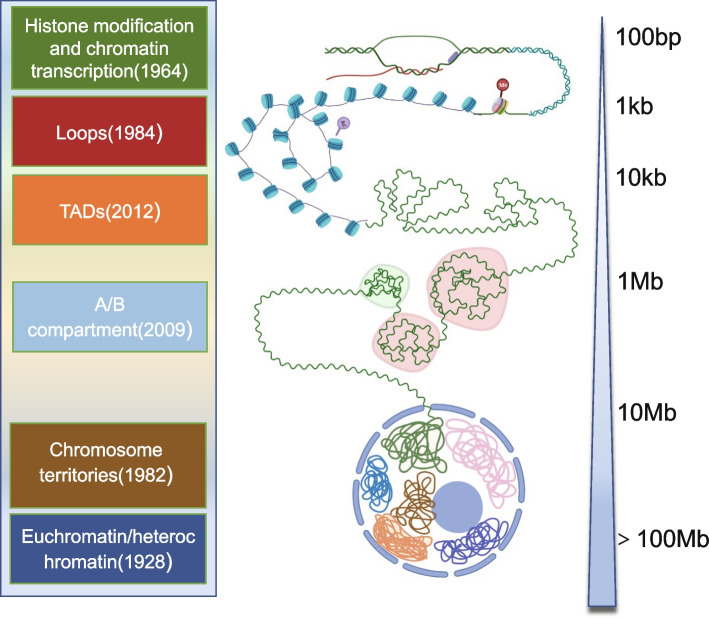
The discovery of main features of chromatin organization Diverse forms of chromatin organization have been identified ranging from the 100 bp scale to more than 100 Mb scale through a combination of different technologies over one century. The association between histone modification and gene transcription was identified through the incorporation of labeled chemical groups into histone structures in 1964 (Allfrey et al. 1964). DNA looping was first discovered using the helical-twist assay (Dunn et al. 1984). With the development of technologies, large chromatin interaction domains called topologically associating domains (TADs) and chromosome compartments indicating the spatial segregation of open and closed chromatin were identified with Hi-C (Dixon et al. 2012; Lieberman-Aiden et al. 2009). Thomas Cremer et al*.* carried out experiments using the laser to confirm the existence of chromosome territories which help to distinguish one chromosome from its neighbors (Cremer et al. 1982). In 1928, Emil Heitz improved cytological staining to define euchromatin and denser heterochromatin (Heitz 1928)

The initial features of genome organization were first observed by Emil Heitz through cytological staining and phase-contrasting to identify heterochromatin and euchromatin in 1928s (Heitz 1928). The development of chromosome-conformation-capture technologies (3C, 4C, 5C, Hi-C, and Micro-C) and their variants have made it possible to examine finer and more comprehensive genomic organizations from territories to compartments, TADs, and even interactive loops (Dekker et al. 2002; Han et al. 2018) (Fig. 1). Previous studies indicated the active transcriptional regions in each chromosomal territory tend to be positioned at the periphery of nuclear speckles, while inactive regions are close to the nuclear envelope (Geyer et al. 2011). Accordingly, the intrachromosomal self-interacting regions can be divided into two types of compartments based on the biochemical marks or activities: A compartment (active marks) and B compartment (inactive marks) (Hildebrand and Dekker 2020). On a finer scale, the active and inactive genomic regions are insulated to form highly interactive TADs with the cooperation of the insulator-binding protein CTCF, cohesin, and others (Beagan and Phillips-Cremins 2020).

Integrated with epigenetic and transcriptomic analyses, the chromatin organization from base pairs to territories has been recognized as an increasingly fundamental and sophisticated aspect of embryogenesis, gametogenesis, lineage commitment, and cell differentiation (Bhattacharya et al. 2009; Dixon et al. 2015; Phillips-Cremins et al. 2013; Sati and Cavalli 2017; Zheng and Xie 2019). The plasticity of chromatin organization is a critical mechanism for the transcriptional regulation of gene expression (Vos 2021). A new study in *Drosophila* revealed that dedicated tethering elements in the genome are critical for fast transcriptional activation by facilitating appropriate enhancer-promoter interactions, while insulators avert unveracious interactions and regulatory interference between neighboring TADs (Batut et al. 2022). The enhancer-promoter interactions are commonly detected during spatiotemporal activation of gene expression. Taking the *sonic hedgehog* (*shh*) limb-bud-specific enhancer MFCS1 as an example, long-range interactions between the *shh* promoter and the MFCS1 enhancer located 1 Mb away were detected in both the anterior and posterior limb buds using 3D-FISH and 3C assays (Amano et al. 2009). The dynamic chromatin conformation of the *shh* locus drives the pulses of *shh* activation. Deletion of MFCS1 eliminates the long-range enhancer-promoter interaction, leading to a loss of limb-specific *shh* expression and truncation of the mouse limb (Amano et al. 2009; Sagai et al. 2005). Further, Hoxd genes have been shown to regulate the induction of *shh* expression in the mouse limb bud (Kmita et al. 2005; Zakany et al. 2004). The precise regulation of Hoxd gene transcription during early mouse limb development was controlled by the opposite and successive actions of two gene deserts flanking this Hoxd cluster on either side (Andrey et al. 2013). In the early phase, the telomeric domain regulates transcription in the proximal limb until a functional and conformational switch occurs toward the opposite topological domain to take over the regulation in the developing distal limb structures (Andrey et al. 2013).

Similarly, genetic mutations that cause alteration of chromatin organization have been found to contribute to the occurrence and progression of various diseases. Previous studies reported that disease-associated enhancer deletion, relocation, and duplication can lead to aberrant rewiring of gene regulatory circuitry between enhancers and their target genes, and consequently lead to pathogenesis (Krijger and de Laat 2016; Nasser et al. 2021). One such example is deletion-, inversion-, or duplication-induced changes in the structure of the TAD-spanning WNT6/IHH/EPHA4/PAX3 locus give rise to pathogenic rewiring of gene-enhancer interactions and eventually limb malformations in humans (Lupianez et al. 2015). Additionally, oncogenes can be activated by genetic mutations that disrupt chromosome neighborhoods in cancer cells (Hnisz et al. 2016). Together, all these important discoveries on chromatin organization suggest that mapping the spatial TADs, their loop interactions, and TAD boundaries can be extremely informative in deciphering the genetic basis of fundamental biological processes. It is broadly recognized that many regulatory mechanisms by which gene transcription is controlled are shared among development, diseases, and regeneration (Bhatt et al. 2014; Wang et al. 2017). Therefore, adopting concepts and methodologies learned from development and diseases should expedite the understanding of how animal regeneration is achieved.

## Genome evolution and the regenerative capacities

The capacity of animal regeneration is unevenly distributed in different animal phyla (Alvarado and Tsonis 2006; Bely 2010; Poss 2010). Animal diversities in nature that involve phenotypic traits, behaviors, and physiology are coded in the genome of each species. Genome evolution can occur at different levels including point mutations, insertion/deletion, genomic recombination, gene duplication, chromosome duplication, and whole genome duplication, and is the driving force for the formation of new features in animals (Dehal and Boore 2005; Henderson and Bomblies 2021; Lin et al. 2019; Lynch and Conery 2000; Tenaillon et al. 2016; Van de Peer et al. 2009). It has been repeatedly observed that evolutionary changes in animal genomes are frequently accompanied by gain or loss of genome size and gene number, expansion or reduction of gene families, and alteration of regulatory complexity (Lynch and Conery 2000; Olson 1999; Petrov 2001; Wittkopp and Kalay 2012). The first analysis of the relationship between genomic features and tissue regeneration was carried out in 1987 by Stanley K. Sessions and Allan Larson (Sessions and Larson 1987). They observed an inverse evolutionary correlation between the genome size and the rate of limb regeneration in salamanders of the family Plethodontidae. Within this largest salamander family, the genome sizes of the group members can range appropriately nine-fold. Interestingly, species with small and large genome sizes in the same phylogenetic group display little differences in the number and shape of the karyotypes (Sessions and Wake 2021). One hypothesis on the evolution of limb regeneration in salamanders is that these animals evolved the ability to regenerate through genome expansion which was mainly driven by the enlargement and dispersion of transposable elements, particularly the LTR retrotransposons (Sessions and Wake 2021). The assembly of the giant axolotl genome (32 Gb, ten times the size of the human genome) was completed recently (Nowoshilow et al. 2018), which provides abundant resources for analyzing the potential genetic regulation of vertebrate regeneration. Massive repetitive sequences (65.6%) were found in the genome and contributed to a dramatic size expansion of introns and intergenic regions compared with those in humans, mice, and frogs (Nowoshilow et al. 2018). Notably, multiple lines of evidence suggest that certain species-restricted coding (*e.g.*, the Ly6 family member Prod1) and non-coding sequences that have been lost or undergone rapid diversification in amniotes contribute to axolotl limb regeneration (Garza-Garcia et al. 2010; Nowoshilow et al. 2018; Silva et al. 2002). In addition to axolotl, genome assembly of other phylogenetically representative species with remarkable regenerative capacities such as the freshwater cnidarian hydra (Chapman et al. 2010), planarians (Grohme et al. 2018), frogs (Kakebeen et al. 2020), zebrafish (Woods et al. 2000), and African killifish (Reichwald et al. 2015; Valenzano et al. 2015) have rendered versatile genomic and transcriptomic analysis for unveiling the mystery of regeneration. Consistently, the presence of a large proportion of noncoding DNA was observed in these regeneration-competent organisms including transposable elements, sequence for the transcription of non-coding RNAs, and other repetitive sequence (Azpiazu and Morata 2022; Harris et al. 2020; Kang et al. 2016; Sen and Ghatak 2015; Shao et al. 2020; Wang et al. 2020c). These observations highlighted that a high percentage of non-coding DNA may be an important source for the generation of new gene regulatory elements that contribute to gene activation upon injury.

**Fig. 2:**
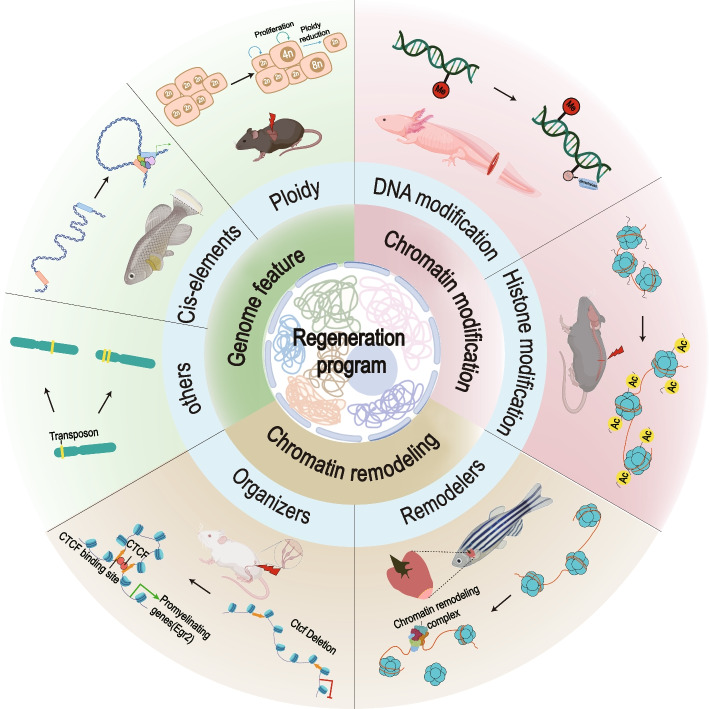
Genome organization and the regulation of tissue regeneration Summary of current understanding on genome organization and the activation of regeneration in different organisms that have been investigated. Genomic elements, genome features, chromatin modifications, and chromatin regulators all contribute to the regulation of animal regeneration

A diploid genome is popular in most animals. Polyploidy, a special condition of possessing more than two complete sets of chromosomes, has been observed to participate during injury response in a variety of tissues like hearts, livers, skeletal, and bone marrows (Fig. 2) (Dornen et al. 2020; Matsumoto et al. 2020; Ovrebo and Edgar 2018). Polyploidization (developmentally programmed or stress-induced) can be achieved through either cell–cell fusion or endoreplication (Ovrebo and Edgar 2018). Such a process can bring certain benefits to cells like enlarged cell size and biomass, which confers enhanced cell longevity due to better tolerance to stress (Anatskaya and Vinogradov 2022). Previous studies demonstrated the transition of diploid cardiomyocytes to polyploid cardiomyocytes attenuates the capacity of cardiac regeneration in neonatal mice and zebrafish due to reduced proliferative potential (Alkass et al. 2015; Gonzalez-Rosa et al. 2018; Kadow and Martin 2018; Kirillova et al. 2021; Yahalom-Ronen et al. 2015). Therefore, it was proposed that polyploidization of cardiomyocytes may underlie the failure of heart regeneration in adult mice. However, polyploid hepatocytes are still capable of cell division and do not weaken the regenerative capacity in mouse liver (Miyaoka et al. 2012). In fruit flies, polyploid cells appear in response to injury in diverse tissues such as intestines and abdominal cuticles, and contribute to the restoration of tissue mass, the maintenance of organ size, the protection against oncogenic insults and genomic stress, and the formation of new diploid cells in regeneration (Bailey et al. 2021; Lucchetta and Ohlstein 2017). The distinct observations in different organs or species suggest further investigations are required for elucidating the contribution of polyploid cells upon tissue damages. Currently, few studies have been reported to characterize the chromatin organization between polyploid and diploid cells during regeneration. However, it has been implicated in plants that polyploidization dramatically enhances the complexity of chromatin structures including changes of A/B compartments and the reorganization of TADs (Garcia-Lozano et al. 2021; Wang et al. 2018).

Further, a recent study using comparative genomics investigated the antler regeneration in ruminants by genome sequencing of pecoran lineages that convergently lack headgear and the collection of hundreds of transcriptomes from bovids and cervids (Lin et al. 2019). This comprehensive analysis indicated that bovid horns and cervid antlers share similar signatures of gene expression and a common neural crest cell origin during development. The rapid regeneration of antlers engages the deployment of oncogenic pathways and a positive selection of certain tumor suppressor genes in deer. Since the first day that multicellular organisms are present on earth, the genome of each extant species is the only information that has been passed from generation to generation during the hundreds of million years of evolution. Therefore, systematic exploration of genome evolution in animals with different regenerative capacities should provide insights into the understanding of the genomic constraints on regeneration.

## Regeneration and the remodeling of chromatin organization

Regeneration requires the activation of a regeneration response program to initiate cell proliferation, migration, differentiation, and other biological processes to restore the lost or damaged organs. All these key processes are accompanied by dynamic remodeling of chromatin organization and transcriptomes of cell populations involved in regeneration (Goldman and Poss 2020; van Steensel and Furlong 2019; Vitulo et al. 2017; Wang et al. 2020a). In addition to the classic regulation of transcription and translation, the epigenetic code has been elucidated to be a key mechanism by which the reconfiguration of genome is achieved to allow the turn-on and turnoff of genes essential for cells to acquire new fates or states (Macchi and Sadler 2020; Moris et al. 2016). Plentiful studies indicated that such epigenetic code involves a complex combination of histone variants, histone modifications, DNA modification, and other factors (Fig. 2) (Rothbart and Strahl 2014; Turner 2007). For instance, histone modifications on certain lysine residues that are conserved from yeast to humans are associated with specific regions of the genome, representing different transcriptional states (Jones 2015; Truong and Boeke 2017). In most cases, active promoters are marked by H3K4me3 and H3K9ac, while the gene body tends to have a higher level of H3K36me3 and H3K79me2/3 for an actively transcribing gene (Gates et al. 2017; Sharakhov and Sharakhova 2015; Slotkin and Martienssen 2007). The *cis*-regulatory elements or enhancers can be defined by H3K27ac (active enhancers) and H3K4me1 (active and primed enhancers) (Nakato and Sakata 2021). Compared with active genes, repressed genes have much higher levels of H3K9me3, H3K27me3, and H4K20me3 (Becker et al. 2016; Peters et al. 2003; Schotta et al. 2004). Whether these histone modifications are functioning as causal effectors is still in debate, such histone marks have been extensively used in the characterization of chromatin accessibility and transcriptional regulation of gene expression under distinct biological contexts.

By taking advantage of these epigenetic markers and tools, a recent breakthrough in understanding the genetic basis of organ regeneration is the discovery of regeneration-responsive enhancers (RREs), also called tissue regeneration enhancer elements or other similar terms (RRE will be used hereafter) (Guenther et al. 2015; Harris et al. 2020; Kang et al. 2016; Sun et al. 2022; Suzuki et al. 2019; Vizcaya-Molina et al. 2018; Wang et al. 2020c; Yang and Kang 2019). Enhancers are DNA-regulatory elements that contain transcription factor binding sites sufficient to activate or boost gene expression by interacting with the gene promoters. The interaction of enhancer-promoter can be achieved by local contacts of DNA-binding transcription factors or by loop formation that mediates the long-range contacts (Higgs 2020). Although it has been suggested that regeneration and development share a similar gene regulatory network (Birnbaum and Sanchez Alvarado 2008; Efroni et al. 2016; Suzuki and Ochi 2020), the mechanisms by which genes are activated can be different. The initial identification of RREs, such as the *lepb*-linked enhancer (*LEN*) responsible for the regeneration-dependent expression of *lepb* in zebrafish (Kang et al. 2016), the WNT gene cluster BRV-B enhancer that directs damaged-induced *wingless* expression in the fruit fly *Drosophila melanogaster* (Harris et al. 2016), a Bmp5 enhancer that activates the endogenous *bmp5* expression following a bone fracture or soft tissue injury in adult mice (Guenther et al. 2015), and the *K-IEN* enhancer that controls the regeneration-induced transcription of *inhba* in African killifish (Wang et al. 2020c), supports the argument that regeneration and development could use distinct regulatory elements to control gene activation. As expected, the regulatory activities of these RREs are turned off or return to a basal level upon the completion of regeneration. Besides, Disruption of certain essential RRE, such as the *K-IEN*, hampers the progression of regeneration (Wang et al. 2020c). Interestingly, a great deal of redundancy seems to be present for RREs because the deletion of multiple RREs identified from different organs or species did not completely block regeneration (Kang et al. 2016; Thompson et al. 2020). This is consistent with what has been observed for many typical enhancers. The regulatory redundancy may function as a strategy to ensure the robustness of regeneration after tissue injury.

The presence of regeneration-dependent regulatory elements in the genome supports that regeneration requires the alteration of chromatin organization to facilitate accessibility for the transcription of regeneration-responsive genes upon injury (Fig. 2). Systematic identification of RREs across different organs and species has revealed a common regulatory mechanism for injury-induced gene expression (Table 1) (Guenther et al. 2015; Harris et al. 2020; Kang et al. 2016; Murad et al. 2021; Sun et al. 2022; Suzuki et al. 2019; Vizcaya-Molina et al. 2018; Wang et al. 2020c; Yang and Kang 2019). Particularly, a side-by-side comparison between zebrafish and African killifish early fin regeneration not only highlighted a conserved regeneration response program (RRP), but also revealed the activation of many previously overlooked species-specific RREs (Wang et al. 2020c). Comparative single-cell RNA-seq analysis confirmed that blastema cells are the major cell population that employs the RRP during regeneration. This teleost defined RRP only contains 49 genes and is triggered by RREs. The widespread activation of species-specific RREs upon injury is quite surprising because zebrafish and African killifish both belong to the teleost fish, share highly comparable cell types in the caudal fin, and demand a similar amount of time for the completion of regeneration after injury. One vivid difference between the two species with ~ 230 million years of evolutionary distance is their life history. Zebrafish is native to freshwater habitats in Southern Asia and inhabit moderately flowing to stagnant water with shallow depth. In contrast, African killifish is found in ephemeral ponds in semi-arid areas subjected to seasonal desiccation and has adapted to a routine drying of living environment by entering diapause as developmentally arrested and desiccation-resistant embryos that remain dormant in the mud (Hu and Brunet 2018). The adaptation of African killifish genome to such a harsh environment has shaped its unique developmental program and may also directly or indirectly contribute to the evolution of the regeneration program (Valenzano et al. 2015; Wang et al. 2020c). As a result, the African killifish *Nothobranchius furzeri* has been implicated as a simpler genetic system for regeneration studies due to the reduced complexity of injury response (Wang et al. 2020c).

**Table 1:** Published datasets regarding chromatin regulation during regeneration

Species	Assay technique	Accession number	Experimental design
Axolotl	ATAC-seq	PRJNA682840	24 samples were collected including eight stages of axolotl limb regeneration: homeostatic, trauma (3 hpa), wounding healing (1 dpa), early-bud blastema (3 dpa), midbud blastema (7 dpa), late-bud blastema (14 dpa), palette stage (22 dpa) and re-differentiated stages (33 dpa) (Wei et al. 2021).
Hofstenia miamia	ATAC-seq	PRJNA515075	*Hofstenia* was amputated transversely and the regenerated tissues at different time points (at 0, 3, 6, 12, 24 and 48 hpa) were harvested (Gehrke et al. 2019).
Mouse	ChIP-seq	GSE104284	The injured and uninjured mice skeletal muscle at nine time points (3 h, 10 h, 24 h, 48 h, 72 h, 168 h, 336 h, 504 h, 672 h) were collected for H3K4me3, H3K4me1, and H3K27ac ChIP-seq (Aguilar et al. 2016).
		GSE61316	Cultured or FACS-sorted mice hair follicle stem cells were collected for H3K27ac, H3K4me1, Crsp1/Trap220, and H3K27me3 ChIP-seq (Adam et al. 2015).
		GSE71134	Percoll density gradient (37% *versus* 70%) was utilized to isolated microglial from injured and sham control spinal cord at 7 days post injury. These microglial were used for H3K4me1 ChIP-seq (Denk et al. 2016).
	ATAC-seq	GSE135406	20 ATAC-seq libraries were generated for sorted Müller glia from retina at multiple time points following two retinal injury models (NMDA treatment for inner retinal injury and light damage for outer retinal injury) (Hoang et al. 2020).
		GSE89928	The wound induced stem cells were sorted from *Sox9*^*CreER*^*;R26YFP* mice with FACS and utilized to establish ATAC-sequencing library (Ge et al. 2017).
		GSE92967	After 6 days, 30 days, and 180 days of IMQ treatment, the epithelial stem cells were purified from treated skin with FACS and utilized for ATAC-seq (Naik et al. 2017).
		GSE158865	Mouse liver bulk ATAC-seq data was generated by harvesting hepatocytes nuclei of undamaged (0 h) and regenerating livers at 48 h, 72 h, and 96 h after PHx (Chen et al. 2020b).
	scATAC-seq	GSE158873	Mouse liver scATAC-seq was performed on freshly isolated hepatocyte nuclei at 0, 48, 72, 96 h after PHx (Chen et al. 2020b).
		GSE153479	Ventricles were collected after injury (myocardial infarction through surgery) at 3 day on P1 and P8 murine hearts for scATAC-seq (Wang et al. 2020d).
Rat	ChIP-seq	GSE63103	The sham control and injured rat P25 sciatic nerve samples were collected after 72 h post injury in rats for H3K27ac ChIP-seq (Hung et al. 2015).
Zebrafish	ATAC-seq	GSE135406	20 ATAC-seq libraries were produced for sorted Müller glia from retina at multiple time points following two retinal injury models (NMDA treatment for inner retinal injury and light damage for outer retinal injury) (Hoang et al. 2020).
		GSE146960	Zebrafish whole-fin ATAC-seq libraries at 0 dpa (freshly amputated), 1 dpa and 4 dpa were generated (Thompson et al. 2020).
	ChIP-seq	PRJNA559885	Zebrafish caudal fins were collected from amputation sites at 0 dpa and 1 dpa for H3K27ac and H3K4me3 ChIP-seq (Wang et al. 2020c).
		GSE158104	Zebrafish regenerative cardiac tissues at 0, 6, 9 dpa were collected for H3K9ac and H3K9me3 ChIP-seq (Wang et al. 2022).
Xenopus laevis	ATAC-seq ChIP-seq	PRJDB9147, PRJDB13124	Proximal and intermediate Pax8:GFP positive nephric tubule cells were collected at day 0 (homeostatic), day 2 (regenerating), and day 5 (regenerated) for ATAC-seq and H3K27ac ChIP-seq (Suzuki et al. 2022).
African killifish	ChIP-seq	PRJNA559885	African killifish caudal fins were collected from amputation sites at 0 dpa and 1 dpa for H3K27ac and H3K4me3 ChIP-seq (Wang et al. 2020c).
Hydra	ATAC-seq	GSE127277	ATAC-seq was generated at different time courses including 0, 2, 4, 6, 12, 24 and 48 h during hydra head regeneration after head dissection (Murad et al. 2021).
	ChIP-seq	GSE127278	H3K4me2, H3K4me3 and H3K27ac ChIP-seq were generated at different time courses including 0, 4, 6, and 24 h during hydra head regeneration after head dissection (Murad et al. 2021).
Drosophila	ATAC-seq ChIP-seq	GSE102841	H3K4me1, H3K27ac, Pol II-8WG16, H3K27me3, Pol II phospho ser5 ChIP-seq were generated with early control and regeneration wing discs samples. For ATAC-seq, regeneration and control samples in different time points (early, mid, and late) were collected to establish ATAC-seq libraries (Vizcaya-Molina et al. 2018).

Summary of datasets generated by histone modification ChIP-seq and ATAC-seq for exploring chromatin dynamics during regeneration in different species

*ATAC-seq:* Assay for Transposase-Accessible Chromatin using sequencing, *ChIP-seq:* Chromatin Immunoprecipitation sequencing, *hpa:* hours post amputation, *dpa:* days post amputation, *IMQ:* imiquimod, *PHx:* partial hepatectomy

Comparative studies of RREs using transgenic reporter assays showed that changes in RREs (*e.g.*, enhancer repurposing and epigenetic silencing) with essential functions during regeneration are an important source for the evolution of regenerative capacities in vertebrates (Harris et al. 2016; Wang et al. 2020c). It was implied that the limited regenerative capacity in Xenopus adult limbs is strongly correlated with the DNA methylation status of a limb-specific *shh* enhancer region during limb regeneration (Yakushiji et al. 2007). This enhancer region is highly methylated in regeneration-incompetent froglets, while is hypomethylated in regeneration-competent tadpoles. Similarly, region-specific epigenetic silencing of a RRE associated with WNT genes limits the regenerative capacity of mature *Drosophila* imaginal discs (Harris et al. 2016). In sum, the activation of species-specific RRE and epigenetic modification of RREs at different development stages argues that certain regeneration-responsive loci in the genome can be subjected to heritable changes in chromatin organization.

## Chromatin regulators and regeneration

The dynamic and strictly controlled regulation of chromatin organization is essential for spatiotemporal and appropriately coordinated gene expression in tissues. Currently, the most characterized chromatin regulators that mediate alterations in the chromatin configuration are DNA modifiers (*e.g.*, methylation and demethylation), histone-modifiers (*e.g.*, methylation, acetylation, ubiquitination, and phosphorylation), ATP-dependent chromatin remodeling complexes (CRCs), and chromatin organizers (*e.g.*, CTCF and cohesin) (Chen and Dent 2014; Clapier et al. 2017; Valencia and Kadoch 2019; Zuin et al. 2014). DNA methylation is a heritable epigenetic mark that cells used to turn off gene expression and this process is directed by DNA methyltransferases (DNMTs). There are five DNMTs encoded in the human genome including DNMT1, DNMT2, DNMT3A, DNMT3B, and DNMT3L. Among those, DNMT1, DNMT3A, and DNMT3B are canonical DNMT enzymes responsible for the addition of methylation marks to genomic DNA (Bestor 2000). In contrast, DNMT2 and DNMT3L lack DNA catalytic activity and are considered non-canonical DNMT members. Genome-wide changes in the pattern of DNA methylation have been observed in different tissues and organs upon injury (Arechederra et al. 2020; Garriga et al. 2018; Gornikiewicz et al. 2016; Lee et al. 2020; Planques et al. 2021; Puttagunta et al. 2014; Yakushiji et al. 2007). Importantly, DNMT1 and the Ubiquitin Like With PHD And Ring Finger Domains 1 (UHRF1) required for localization and stability of DNMTs are key players in the maintenance of methylation during cell proliferation (Bronner et al. 2019). It is not unexpected that these proteins are involved in liver, axon, and muscle regeneration (Lee et al. 2020). Nevertheless, a study in zebrafish revealed that the patterns of lineage-specific DNA methylation are stably maintained during fin regeneration and RREs are preset as hypomethylated before tissue damage (Lee et al. 2020). This result suggested that regeneration responsive genes under the control of RREs are likely activated independent of the DNA demethylation during fin regeneration (Lee et al. 2020; Wang et al. 2020c).

Histone modifiers, such as histone acetyltransferases (HATs) and deacetylases (HDACs) are widely implicated in the regulation of regeneration (Friedrich et al. 2019; Gordon et al. 2015; Huynh et al. 2017). For example, the HAT p300 could acetylate histone H3, pro-regenerative transcription factor p53, and CCAAT-enhancer binding proteins to activate a silent gene regulatory program that is sufficient to promote rat intrinsic axonal regeneration (Gaub et al. 2011). The inhibition of HDACs appears to be a powerful strategy for promoting regeneration in different systems (Ahmad Ganai et al. 2016; Flici and Frank 2018; Huynh et al. 2017). Remarkably, Müller glia specific overexpression of a pro-neural transcription factor Ascl1, in combination with a histone deacetylase inhibitor, enables adult mice to regenerate neurons from Müller glia upon retinal injury (Jorstad et al. 2017). In addition, the motif of the histone demethylase ARID3A was commonly found in RREs (also named regeneration signal-response enhancers) identified in regenerating Xenopus nephric tubules (Suzuki et al. 2019). ARID3A was recruited to reduce the repressive H3K9me3 levels on RREs to promote cell cycle progression and the outgrowth of nephric tubules (Suzuki et al. 2019).

ATP-dependent chromatin remodelers control chromatin architecture by directly mobilizing nucleosomes to enhance local chromatin accessibility (Piatti et al. 2011). Four important families of chromatin remodelers that are conserved from yeast to humans were identified including SWI/SNF, imitation switch (ISWI), nucleosome remodeling and deacetylase (NuRD), and inositol requiring 80 (INO80) complexes (Clapier and Cairns 2009; Tyagi et al. 2016). A recent in vivo CRISPR screening identifies the ISWI component BAZ2 as a druggable suppressor of mammalian liver regeneration (Jia et al. 2022). Inhibition of BAZ2 accelerates liver regeneration through increased ribosomal components and protein synthesis, indicating that targeting chromatin remodelers can be permissive to promote cell growth (Jia et al. 2022). Further, two SWI/SNF complexes, PBAP and BAP, control two distinct aspects (growth and cell fate) of regeneration, respectively (Tian and Smith-Bolton 2021). The PBAP complex is responsible for regenerative growth and developmental timing, while the BAP complex is in charge of correct patterning and cell fate. Additionally, Brg1, another member of the SWI/SNF complex, was reported to regulate myocardial proliferation and regeneration in zebrafish by repressing cyclin-dependent kinase inhibitors (Xiao et al. 2016).

Chromatin organizers, such as CTCF and cohesin, function as physic hinges in organizing TADs into architectural loops (Chien et al. 2011; Han et al. 2008). These proteins participate in higher levels of regulation in chromatin organization. Cohesin catalyzes genome folding through loop extrusion which stops at the CTCF binding sites with a convergent orientation (Tang et al. 2015). The dynamic loop formation increases spatial *cis*-tethering over long distances and promotes transcriptional regulation. CTCF was identified with increased expression during cell reprogramming, which helps repress somatic genes and maintain chromatin accessibilities for partial enhancer-promoter interactions in cooperation with an ISWI chromatin remodeler SMARCA5 (Song et al. 2022). Moreover, a dual role of CTCF-dependent chromatin organization in controlling myelinogenic programs and recruiting chromatin-repressive complexes was reported in Schwann cells (Wang et al. 2020b). Deletion of *CTCF* blocks the interaction between promoter and enhancers of the locus of *Egr2*, leading to a strong reduction in the expression of the pro-myelinogenic factor EGR2 and the suppression of Schwann cell differentiation during nerve repair. Therefore, global changes of chromatin organization caused by aberrant regulation of chromatin organizers can cause unpredicted transcriptional and functional alterations in cells. In summary, chromatin regulators play a significant role in remodeling the architecture of chromatin and are actively involved in the regulation of regeneration (Fig. 2). Understanding how these chromatin regulators establish accessible chromatin and select enhancer-promoter interactions after injury should be informative in developing new strategies for re-activating regeneration in damaged human organs.

## The AP-1 complex is a potential master regulator of the regeneration response program

**Fig. 3:**
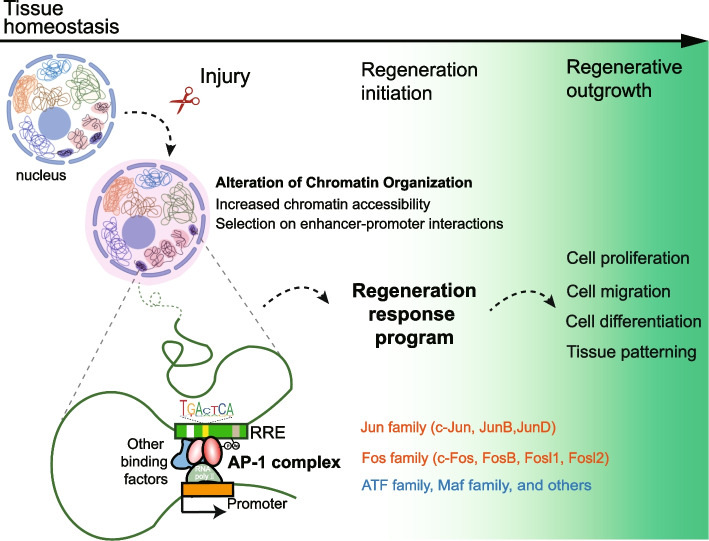
AP-1 complex and the activation of the regeneration response program A model for the activation of the regeneration response program (RRP). The AP-1 complex is a potential master regulator of the RRP. Upon tissue damage, alteration of chromatin organization is initiated to establish accessible chromatin. Combined with other binding factors, the AP-1 complex selects enhancer-promoter interactions to turn on the RRP, which leads to the initiation and progression of regeneration

Despite RRP is subjected to evolutionary changes and contains species-specific components, its regulation seems to share common features (Wang et al. 2020c). Identification of the master regulator of RRP is one of the key tasks in the field of regeneration. In adult mice, activation of the neuregulin1 (*Nrg1*) pathway induces cell-cycle reentry for cardiomyocytes and promotes myocardial regeneration, resulting in improved heart function post myocardial infarction (Bersell et al. 2009). Furthermore, transgenic reactivation of *Nrg1* expression in intact zebrafish hearts turns on many hallmarks of cardiac regeneration and significantly enhances ventricular size (Gemberling et al. 2015). Thus, *Nrg1* was considered an injury-induced mitogen of cardiomyocytes with the power to induce endogenous heart regeneration program in zebrafish (Gemberling et al. 2015). Because regeneration-responsive genes are restricted at the basal transcriptional levels during homeostasis, activation of this class of genes requires the remodeling of chromatin. Recently, the transcription factor binding motif of the Activator Protein 1 (AP-1) complex was found to be present in reported RREs including *LEN*, *BRV-B*, *K-IEN*, and others (Goldman and Poss 2020; Harris et al. 2016; Harris et al. 2020; Kang et al. 2016; Tamaki et al. 2023; Thompson et al. 2020; Wang et al. 2020c). The AP-1 complex, assembled through the dimerization of the bZIP domain between the Fos and Jun subunits, mediates many cellular and physiological functions in development and diseases. Remarkably, comparative motif enrichment analysis identified the presence of AP-1 binding motif as a common feature in RREs identified from both African killifish and zebrafish fin regeneration (Wang et al. 2020c). Deletion of the AP-1 motifs in tested RREs led to a complete loss of enhancer activities upon tissue damage (Wang et al. 2020c). Moreover, the AP-1 motifs recognized by the Jun family proteins (Jun, JunB, and JunD) exhibit a higher frequency in highly regenerative fish genomes than in human and mouse genomes (Wang et al. 2020c). To date, the AP-1 complex has been shown to play essential roles in regulating fin regeneration, *Xenopus* tail regeneration, zebrafish heart regeneration, axolotl spinal cord regeneration, mice liver regeneration, peripheral nerve regeneration, and skin repair (Angel et al. 2001; Beisaw et al. 2020; Ishida et al. 2010; Nakamura et al. 2020; Patodia and Raivich 2012; Sabin et al. 2019; Stepniak et al. 2006). Interestingly, the AP-1 complex directs enhancer selection to govern precise gene expression so that cells can differentiate and acquire specialized functions (Bejjani et al. 2019). Such enhancer selection is determined by a collaborative binding of FOS/JUN and cell-type-specific transcription factors to enhancers and the recruitment of the SWI/SNF (BAF) complex to create accessible chromatin (Vierbuchen et al. 2017). This is consistent with the observation that AP-1 transcription factors control the cardiomyocyte response to cryoinjury by regulating chromatin accessibility (Beisaw et al. 2020). These injury-induced open chromatin regions with AP-1 binding motifs allow the activation of a regeneration program that facilitates cardiomyocyte dedifferentiation, proliferation, and protrusion into the damaged area. All these data point out that AP-1 complex may function as a master regulator in the activation of RRP by controlling enhancer selection and chromatin accessibility (Fig. 3).

Whether AP-1 complex alone is sufficient to activate RRP needs further investigation. It has been noted that regeneration of different organs involves both shared and organ-specific regulators (Hui et al. 2017; Iismaa et al. 2018). Therefore, AP-1 complex may function with tissue-specific transcription factors and chromatin regulators to initiate regeneration upon tissue damage. Additionally, in highly regenerative invertebrates, such as acoel, the gene *early growth response* (*egr*) was identified using ATAC-seq as a pioneer factor to regulate early wound-induced genes (Gehrke et al. 2019). In vertebrates, *egr-1* has been considered a critical mediator of fibroblast activation and fibrotic response triggered by diverse stimuli (Bhattacharyya et al. 2013). Abnormal activation of *egr-1* has been linked to fibrosis and human fibrotic disorders (Bhattacharyya et al. 2011). Such differences may suggest basal animals with whole-body regeneration and vertebrates that only retain regenerative capacities in certain tissues or organs deploy distinct master regulators to initiate RRP. It would be interesting to investigate the major regulatory differences between animals with unlimited regenerative capacities and others with limited regeneration.

## Conclusions

As a long-standing question in biology, regeneration has been widely investigated at different levels including cell regeneration, tissue/organ regeneration, appendage or structure regeneration, and whole-body regeneration. Numerous studies derived from distinct organs and species indicate that mechanisms established from a single species do not ensure successful application in humans. Therefore, there is an urgent need for the identification and characterization of conserved and species-specific regeneration response programs. Synthesizing information collected from different organisms ranging from the basal animals, such as sponges and hydra, to mammals such as deer and African spiny mice is critical for establishing evolutionarily conserved mechanisms underlying regeneration. Further, increasing attention has been paid to develop tools for the manipulation of gene expression in damaged tissues to reactivate regeneration. One such promising tool is the RRE or tissue-regeneration enhancer elements that can confer spatial or temporal control of key regeneration genes (Kang et al. 2016). A new study from the Poss group demonstrated that zebrafish RREs were sufficient to stimulate or suppress endogenous gene expression after ischemic myocardial infarction in mice (Yan et al. 2023). Interestingly, a constitutively active YAP factor driven by such tool was sufficient to promote cardiac regeneration in mice, resulting in improved function of the injured heart (Yan et al. 2023). In addition, other tools (such as chromatin-modifying drugs and metabolites and other small molecules) that are lack of context specificity have also been used to stimulate regeneration in mouse model. Further development and optimization of these tools will pave the way for establishing reliable strategies to restore regeneration in regenerative medicine.

Chromatin organization dependent gene regulation is a highly conserved regulatory mechanism that can be applied in development, regeneration, and diseases. Although several regulators controlling chromatin accessibility during regeneration have been identified, many fundamental questions remain elusive. For example, we still don’t know 1) what kind of chromatin organization underlies regenerative competency? 2) what factors are sufficient to establish such chromatin organization upon tissue damage? 3) what are the differences in chromatin organization between animals with unlimited regenerative capacities and others with limited regeneration? and 4) how do epigenetic modifications and regulations fine-tune regeneration in different species? New methodologies and technologies that have been developed for examining chromatin organization with high resolution will facilitate addressing these questions in future studies. Particularly, the combination of single-cell chromatin profiling techniques (*eg.,* scATAC) and single-cell omics (*eg.,* scRNAseq) provides new opportunities for identifying differences in gene expression and chromatin accessibility in each cell population involved in tissue regeneration (Chen et al. 2020b; Sinha et al. 2022; Wang et al. 2020d). For example, a recent integrated single-cell RNA-seq and ATAC-Seq analysis systematically mapped cell state transitions in more than 10,000 hepatocytes during liver regeneration and identified injury-associated signaling pathways involved in transitioning hepatocytes (Chen et al. 2020b). Similarly, another analysis helped generate open chromatin landscapes and regeneration-associated gene regulatory networks of distinct cardiac cell types following myocardial infarction (Wang et al. 2020d). Mapping cell type specific chromatin organization during regeneration is a critical step toward understanding the genetic basis of regeneration and the uneven distribution of this feature in animals.

## Data Availability

Not applicable.

## Abbreviations

3C: Chromosome Conformation Capture
CRCs: ATP-dependent chromatin remodeling complexes
DNMT: DNA methyltransferase
HAT: Histone acetyltransferase
HDAC: Histone deacetylase
HDM: Histone demethylase
HMT: Histone methyltransferase
UHRF1: Ubiquitin Like With PHD And Ring Finger Domains 1
INO80: Inositol requiring 80
NuRD: Nucleosome remodeling and deacetylase
ISWI: Imitation Switch
RRE: Regeneration-Responsive Enhancer
SWI/SNF: Switching defective/Sucrose nonfermenting
TADs: Topologically Associating Domains
